# Persistence of stigma and the cessation of substance use: comparing stigma domains between those who currently use and those who no longer use substances

**DOI:** 10.3389/fpsyt.2023.1308616

**Published:** 2024-01-08

**Authors:** Krishna Patel, Emily Pokorski, Donna Norkoli, Emily Dunkel, Xinyue Wang, Lawrence H. Yang

**Affiliations:** ^1^National Association of County and City Health Officials, Washington, DC, United States; ^2^District Health Department #10, Cadillac, MI, United States; ^3^School of Global Public Health, New York University, New York, NY, United States; ^4^Teachers College, Columbia University, New York, NY, United States; ^5^Mailman School of Public Health, Columbia University, New York, NY, United States

**Keywords:** substance use disorder, internalized stigma, cessation, stigma, persistence of stigma

## Abstract

**Introduction:**

Cessation of substance use, a facet of recovery, as well as mitigating stigma experienced by individuals with substance use disorder (SUD), are important to supporting health and well-being of those who use substances. But there is limited and mixed evidence on whether cessation of substance use has a positive impact on individuals’ stigma experiences. This study examined whether there were differences in stigma perceptions between those who self-report using substances and those who self-report not currently using substances associated with their SUD.

**Materials and methods:**

A survey was conducted among individuals in 10 counties of Michigan with self-identified history with SUD. The survey aimed to understand five dimensions of stigma perceptions: enacted stigma, anticipated stigma, internalized stigma, social withdrawal, and treatment stigma. Survey items for each measure were adapted from prior literature. The mean was calculated for each stigma measure for analyses. Data analyses tested whether there were significant differences in each of the five stigma domains between the two groups using either regression or *t*-test, depending on the necessity to include covariates.

**Results:**

Findings suggested that among the five stigma domains, only internalized stigma showed statistically significant differences between the two groups (*b* = 0.19, se = 0.08, *p* < 0.05) after adjusting for covariates (as needed). Those who were no longer using substances had lower internalized stigma compared to those who were currently using substances associated with their SUD. Analyses suggested that the other four stigma domains, enacted stigma, anticipated stigma, social withdrawal, and treatment stigma, did not show statistically significant differences between the two groups.

**Discussion:**

While self-stigmatization (i.e., internalized stigma) was lower among those who report no longer using, our patterns suggest a persistence of stigma regardless of cessation of substances associated with SUD, particularly among stigma domains that are based on perceptions of how others may still perceive individuals who have used substances. Though more research is needed, results suggest that public health programmatic, policy, and campaign efforts that aim to eliminate stigma should account for and tailor to both people who report using and those who report no longer using substances to capture the breadth of needs in communities.

## 1 Introduction

According to the 2021 National Survey on Drug Use and Health (NSDUH), 43.6 million Americans aged 12 or over – approximately 16.5% of the national population – met the DSM-5 criteria for having a substance use disorder (SUD) (1). Since the start of the COVID-19 pandemic, an increase has been observed in substance use and drug overdose in the U.S. (2). In addition, opioid use disorder (OUD) and opioid addiction remain at epidemic levels and continue to increase in the U.S. and worldwide (3). Stigma is strongly associated with SUDs and OUD (4), and has been found to be higher than stigma associated with other mental illnesses and physical disorders that could lead to disabilities (5, 6). Consequently, the National Institute of Drug Abuse (7) has most recently prioritized stigma research in their plan to address rising rates of SUDs and overdose in the United States.

Stigma plays a substantial role in the experience of SUD. Public stigma toward individuals with SUDs is high (8), as is self- or internalized stigma and structural stigma (9–11). Stigma impedes individuals’ help-seeking, treatment entry, and treatment adherence for SUD (12), as well as leads to adverse mental health consequences (8, 13). While stigma impacts treatment and recovery outcomes, it is not clear whether stigma becomes mitigated if individuals no longer report using substances (i.e., cessation of substance use). Because definitions of recovery from SUD (while multifaceted) often feature reduction or cessation of substance use as a key component (14), the key aim of this paper was to examine this key aspect of recovery—the cessation of use of substances —and its association with stigma.

The impact of stigma on individuals with SUDs and their recovery remains under-examined, particularly as it relates to how stigma may change with the cessation of substance use (15). Findings regarding the relationship between stigma and the status of an individual’s substance use are mixed, and stigma may persist even after individuals report no longer using substances. A seminal study conducted by Link et al. (16) examined varying domains of stigma (i.e., enacted, perceived, and social stigma) among a group of men with SUD and mental illness and found no significant relationship between the cessation of substance use and stigma. In addition, there are few existing studies that examine stigma after the cessation of substance use; these studies are mainly qualitative and also show mixed findings. In a qualitative study examining the impacts of substance use treatment on reducing stigma after release from incarceration, participants indicated that the cessation of their substance use and success in recovery impacted the perceptions and stigmatizing attitudes of others, citing reduced discrimination and increased trust (17). Further, participants highlighted that internalized stigma decreased with treatment, citing a positive clinical environment in non-judgmental relationships with providers and positive connections with peers. Interviews conducted by Matthews et al. (18) support an association between reduction in self-stigma and recovery, with participants reporting that lower levels of shame and guilt were associated with their cessation of substance use.

Kulesza et al. (19) found, in a small quantitative longitudinal study with individuals attending intensive inpatient treatment (*n* = 17), that participants who reported higher self-stigma following treatment also reported more frequent substance use at one-month follow-up. In-depth interviews (*n* = 22) conducted by Romo and Obiol (20) showed that participants in recovery felt the impacts of stigma (e.g., decreased social support and heightened isolation) were factors in resuming substance use. Finally, Burgess et al. (21) found via focus groups with individuals in recovery that some participants’ experiences of stigma made staying in recovery difficult and prompted some individuals to resume their former substance use. However, for other participants, stigma motivated them to stay substance-free, citing a desire to prove others wrong.

Due to the limited and inconsistent findings between stigma and cessation of substance use and the restricted (i.e., primarily qualitative) methodologies presented in the existing literature, this study aimed to quantitatively examine, utilizing a community-based sample, whether there were differences in stigma for individuals who reported no longer using substances associated with their SUD. This study thus contributes to an emerging literature on stigma in populations no longer using substances associated with their SUD.

## 2 Materials and methods

### 2.1 Survey design and participants

This study surveyed a community-based sample of individuals with history of substance use disorder (SUD) in Michigan utilizing convenience-sampling methods. The survey focused on understanding perceptions of stigma among individuals with SUD, both those who reported currently using substances related to their substance use disorder and those who reported being free of using substances related to their disorder.

Individuals were eligible if they were 18 years or older, had a self-reported history with a substance use disorder, and lived in one of the following counties in Michigan: Antrim, Benzie, Charlevoix, Emmet, Grand Traverse, Kalkaska, Leelanau, Manistee, Missaukee, Wexford. These 10 counties were selected based on their recent statistics reflecting substance use and overdose (22). Three of the ten counties (Kalkaska, Manistee, and Wexford) had a 3-year (2018–2020) average nonfatal overdose emergency department visit rate per 100,000 that was greater than the overall rate in the state of Michigan. In 2020, six out of the ten counties (Antrim, Benzie, Charlevoix, Kalkaska, Manistee, and Wexford) had a higher opioid prescription unit rate per 1,000 residents than the state of Michigan overall. The drug-related arrest rate per 100,000 residents for Benzie, Kalkaska, Manistee, Missaukee, and Wexford was greater than that found in the state overall (2020). Additionally, in 2021, four out of the ten counties (Antrim, Emmet, Kalkaska, and Leelanau) had a smaller percent of their population living within a 30-min drive of an SUD treatment center compared to the state of Michigan overall (22).

The survey was sent to 25 community leaders, representing 30 organizations, working within various hospitals, recovery houses, non-profits, coalitions, and peer recovery groups within these 10 counties to share with their networks. Surveys were distributed primarily electronically via Alchemer, an online survey platform, but paper copies were made available as needed. Social media posts with the survey link attached were distributed broadly on District Health Department #10’s social media pages (i.e., Facebook and Instagram). Surveys were confidential, and no personal identifying information was required. After completing the survey, participants had the option to fill out a separate form to enter personal information for a $50 gift card raffle.

This study collected 343 responses between October 18 and November 14, 2022. This project was reviewed by the institutional review board at Michigan Department of Health and Human Services. Because of the broad scope given to public health agencies (i.e., including public health surveillance, which this survey falls under), a determination notice was issued indicating that this project fell under “Not Human Subjects Research.”

### 2.2 Measures

The survey included five domains of stigma adapted from prior measures and studies: enacted stigma, internalized stigma, and anticipated stigma (Substance Use Stigma Mechanisms Scale) (23), social withdrawal (Internalized Stigma of Substance Abuse Scale) (24), and treatment stigma (Treatment Stigma) (25), which were used as outcomes in analyses (See Table 1 for survey items and definitions). Additional items were added or adapted to the survey as needed (e.g., an item assessing current use of substances related to the individual’s SUD). Means across stigma measures were calculated, which allowed the summed measure to be on the same response scale as the original items. Items were reverse coded as needed to indicate increasing levels of stigma.

**Table 1:** Stigma domains, definitions, and corresponding survey items.

Measure	Definition^a^	Items	Response options^b^	Cronbach’s alpha^c^
Enacted stigma	“A direct experience of discrimination and rejection from members of the larger society” (26).	Family members have thought that I cannot be trusted. Employers have thought that I am still using drugs. Healthcare workers have not listened to my concerns. Law enforcement have treated me poorly due to my substance.	Never (1), not often, somewhat often, often, very often (5)	0.62
Anticipated stigma	“A process whereby stigmatized individuals think that most people believe common negative stereotypes about individuals belonging to the same stigmatized category as they do” (26).	Family members will treat me differently. Employers will treat me poorly. Healthcare workers will give me poor treatment. Healthcare workers will think that I’m pill shopping, or trying to con them into giving me prescription medications to get high or sell to others. Law enforcement will think that I cannot be trusted.	Never (1), not often, somewhat often, often, very often (5)	0.67
Internalized stigma	“Negative thoughts, feelings, and diminished self-image resulting from identification with the stigmatized group and anticipation of rejection from the larger society” (26).	I feel I’m not as good as others because I have a substance use disorder. I feel ashamed of having a substance use disorder. Having a substance use disorder makes me feel unclean.	Strongly disagree (1), disagree, agree, strongly agree (4)	0.64
Social withdrawal	“Limiting social interaction to those who know about and tend to accept one’s stigmatized condition” (27).	Being around people who do not have a substance use disorder makes me feel out of place or inadequate. I stay away from social situations in order to protect my family or friends from embarrassment. Negative stereotypes about substance use keeps me isolated from the “normal” world.	Strongly disagree (1), disagree, agree, strongly agree (4)	0.60
Treatment stigma	“Stigmatizing attitudes about receiving treatment…, which in clients presenting for treatment would also reflect internalized shame…” (28).	People will see a person in a less favorable way if they come to know he/she received treatment for substance use.	Strongly disagree (1), disagree, agree, strongly agree (4)	N/A

^a^ Definitions used in this study reflect the ones in the articles referenced. The term anticipated stigma is used in this study which is referenced as perceived stigma in the reference article, though definitions are applied similarly.

^b^ Numerical response options presented are after reverse coding for analysis.

^c^ Cronbach’s alpha’s calculations based on final analytic sample *N* = 329 and listwise deletion across items in the measure.

The main independent variable was a binary measure of self-reported cessation of current use of substances, based on the item: *Are you currently free of using any substances that were previously associated with your substance use disorder? (yes, no, prefer not to respond)*. Those who indicated *prefer not to respond* were dropped from the analyses (*N* = 14) for a final analytic sample of *N* = 329.

### 2.3 Statistical analysis

Statistical analyses were conducted in Stata 17 (29). Statistical significance was based on *a priori* alpha level of 0.05. Chi-square tests across key demographics were used to determine whether there were significant differences between the two groups: those who currently identified as using substances and those who did not. If any demographic characteristics were significantly different across the two groups, a one-way ANOVA was conducted to test whether the demographic item was significantly associated with any of the five stigma outcome measures. If not significantly associated, a student’s t-test or non-parametric equivalent (Mann–Whitney/Wilcoxon rank-sum) test was run to test differences in stigma (for each measure independently) across the two groups. If demographic characteristics were significantly associated with the stigma outcome measure, independent regression analysis was conducted instead to include those items as covariates.

## 3 Results

Demographic characteristics of participants are presented in Table 2. Across the 329 participants, most identified as male (61%) and were between the ages of 25 and 44 (82%). Almost all participants identified as White (90%). Education and income were more varied; almost one in four had a bachelor’s degree and 16% had completed up to high school diploma/GED only. Almost half of the participants reported an income of between $130,000 and $149,999 and approximately a quarter reported an income of under $50,000.

**Table 2:** Descriptive characteristics of sample (*N* = 329).

Demographics	Overall *N* (%)	Free of substances *N* (%)	Not free of substances *N* (%)
Gender *chi* (2) = 23.31; *p* < 0.001			
Male	200 (61%)	113 (52%)	87 (79%)
Female	125 (38%)	103 (47%)	22 (20%)
Nonbinary or Transgender	4 (1%)	3 (1%)	1 (1%)
Age *chi* (5) = 8.02; *p* = 0.16			
18 to 24 years	8 (2%)	7 (3%)	1 (1%)
25 to 34 years	155 (47%)	99 (45%)	56 (51%)
35 to 44 years	118 (36%)	76 (35%)	42 (38%)
45 to 54 years	27 (8%)	21 (10%)	6 (5%)
55 to 64 years	13 (4%)	8 (4%)	5 (5%)
65 years or older	8 (2%)	8 (4%)	0 (0%)
Race/Ethnicity *chi* (6) = 4.77; *p* = 0.57			
White	297 (90%)	194 (89%)	103 (94%)
Black	5 (2%)	3 (1%)	2 (2%)
Native American or Alaska Native	17 (5%)	15 (7%)	2 (2%)
Asian	1 (0.3%)	1 (0.5%)	0 (0%)
Native Hawaiian or other Pacific Islander	4 (1%)	3 (1%)	1 (1%)
Hispanic/Latinx	3 (1%)	2 (1%)	1 (1%)
Prefer not to respond	2 (1%)	1 (0.5%)	1 (1%)
Education *chi* (7) = 30.13; *p* < 0.001			
Some high school	19 (6%)	15 (7%)	4 (4%)
High school diploma or GED	34 (10%)	29 (13%)	5 (5%)
Some college	41 (12%)	35 (16%)	6 (5%)
Trade school	1 (0.3%)	1 (0.5%)	0 (0%)
Associate degree	64 (19%)	33 (15%)	31 (28%)
Bachelor’s degree	89 (27%)	58 (26%)	31 (28%)
Some graduate school	55 (17%)	27 (12%)	28 (25%)
Master’s degree or higher	26 (8%)	21 (10%)	5 (5%)
Income *chi* (6) = 51.78; *p* < 0.001			
$0 to $19,999	23 (7%)	16 (7%)	7 (6%)
$20,000 to $49,999	60 (18%)	53 (24%)	7 (6%)
$50,000 to $89,999	59 (18%)	50 (23%)	9 (8%)
$90,000 to $129,999	19 (6%)	17 (8%)	2 (2%)
$130,000 to $149,999	157 (48%)	75 (34%)	82 (75%)
$150,000+	9 (3%)	7 (3%)	2 (2%)
Prefer not to answer	2 (1%)	1 (0.5%)	1 (1%)

Overall *N* = 329; free of substances *N* = 219 (67% of overall); not free of substances *N* = 110 (33% of overall). Not displayed in the table: 0% for other/prefer not to respond for gender.

Between the two groups, self-report of being free of substances and those not reporting being free of substances, there were several differences. Race/ethnicity and age were not significantly different across the two groups. Gender, education, and income did show significant differences across the two groups. There were higher percentage of males among those who were not free of substances (79%) compared to those who were free of substances (52%). Income tended to be higher among those reporting not being free of substances with 75% reporting income of $130,000 to $149,999; in contrast, there was more variability among those who reported being free of substances.

The means of the stigma measures are presented (Table 3) and appeared greater than the mid-point of their respective ranges, suggesting a relatively high amount of stigma among the sample overall. Generally, those who reported not being free of substances appeared to have higher mean values of stigma. A t-test was run for the treatment stigma outcome only as neither gender, race, nor income were associated with that measure, while regression was used for other stigma measures based on ANOVA results of relationships between covariates and each stigma outcome. For covariate inclusion, gender and income were included in all four stigma measures, and education was included only in the internalized stigma model.

**Table 3:** Self-stigma among those free or not free of substances associated with their SUD.

Measure (Increasing self-stigma)	Mean (SD)	Mean (SD) *free of substances*	Mean (SD) *Not free of substances*
Enacted stigma (range = 1–5; *N* = 326)	3.10 (0.85)	3.06 (0.90)	3.18 (0.73)
Anticipated stigma (range = 1–5; *N* = 322)	3.14 (0.77)	3.06 (0.81)	3.30 (0.67)
Internalized stigma (range = 1–4; *N* = 324)	2.71 (0.71)	2.58 (0.73)	2.98 (0.59)
Social withdrawal (range = 1–4; *N* = 324)	2.59 (0.66)	2.49 (0.66)	2.79 (0.61)
Treatment stigma (range = 1–4; *N* = 323)	2.83 (0.82)	2.83 (0.83)	2.83 (0.80)

Four stigma measures were run as outcomes in regression as there were covariates significantly associated with both being free of substances and the outcome measure.

For treatment stigma, there was no statistically significant difference across the two groups of participants (*t*(321) = 0.02; *p* = 0.99). Regression analyses for the remaining four stigma measures are presented in Table 4. After controlling for demographic covariates, there was a significant relationship between reported substance use and internalized stigma only. Those who reported not being free of substances (i.e., currently reported using) had significantly greater internalized stigma compared to those who reported being free of substances (coef = 0.191; robust SE = 0.076; 95% CI = 0.041–0.340; *p* = 0.013). After controlling for demographic covariates in each model, there were no significant differences between the groups that reported being free and not free of substances on the three stigma domains of enacted stigma, anticipated stigma, or social withdrawal.

**Table 4:** Regression models relationship between free of substances and different stigma measures.

Model/Outcome	Anticipated stigma	Enacted stigma	Internalized stigma	Social withdrawal
	coef (se)	coef (se)	coef (se)	coef (se)
Free of substances (no) Reference: (1)/yes	−0.002 (0.082)	−0.155 (0.089)	0.191* (0.076)	0.067 (0.074)
Reference: Male				
Female	−0.288 (0.148)	−0.587*** (0.162)	−0.044 (0.125)	−0.251* (0.108)
Nonbinary or transgender	−0.435 (0.448)	−0.702 (0.570)	−1.036*** (0.261)	−0.493* (0.217)
Reference: $0 to $19,999				
$20,000 to $49,999	0.059 (0.214)	−0.032 (0.214)	−0.109 (0.187)	0.080 (0.164)
$50,000 to $89,999	0.147 (0.221)	−0.265 (0.211)	−0.042 (0.188)	−0.131 (0.172)
$90,000 to $129,999	−0.141 (0.242)	−0.430 (0.265)	−0.155 (0.239)	−0.174 (0.218)
$130,000 to $149,999	0.427 (0.222)	0.051 (0.225)	0.467* (0.207)	0.362* (0.165)
$150,000+	−0.753 (0.463)	−0.798 (0.484)	−0.376 (0.304)	−0.293 (0.363)
Prefer not to answer	−0.550 (0.351)	−0.674*** (0.191)	−1.273** (0.435)	−1.140*** (0.182)
Reference: High school or less				
More than HS, but less than Bachelors			−0.258* (0.128)	
Bachelors			−0.141 (0.142)	
Greater than Bachelors			−0.238 (0.143)	
N	322	326	324	324
adj. *R*-sq	0.18	0.18	0.25	0.26

* *p*<0.05, ** *p*<0.01, *** *p*<0.001; se = robust standard error; coef = unstandardized beta coefficient. Education was collapsed into four categories for anova and regressions – high school or less, more than high school but less than bachelor’s degree, bachelor’s degree, and more than bachelor’s degree.

## 4 Discussion

This study’s primary findings indicate a difference in the experiences of stigma between individuals who report no longer using substances versus those who report currently using substances in one sole stigma domain, internalized stigma. A significant decrease on items that measured participants’ own stigmatizing perception of themselves (e.g., *“I feel I’m not as good as others because I have a substance use disorder”*) suggests that the cessation of substance use is associated with the specific domain of self-stigma (or internalized stigma). An association between self-stigma and substance use is consistent with prior findings from Moore et al. (17), Matthews et al. (18), and Kulesza et al. (19), who reported that decreased stigma and shame as well as increased self-acceptance can forestall future substance use, and that increased self-stigma is positively correlated with frequency of substance use following treatment, respectively.

In contrast, the remaining four domains of stigma (enacted stigma, anticipated stigma, social withdrawal, and treatment stigma) did not show statistically significant differences between groups after controlling for covariates (as needed), which aligns with studies such as Link et al. (16) that also found no significant change in domains of stigma with the cessation of substance use. A lack of differences in these four stigma domains suggests that stigma items that assessed participants’ perception of how *others* might still perceive them (e.g., “*Employers have thought that I am still using drugs”*) did not differ even if individuals reported current cessation of substance use. Given the chronic nature of relapse in SUD, it is possible that others may not readily believe individuals who report a stoppage of substance use. This is evidenced by results from Moore and colleagues (17), who found that participants reported difficulty rebuilding trust in their interpersonal relationships following initiation of substance use problems. Their findings suggest that reduction in forms of stigma based on others’ perceptions of the individual may occur over prolonged periods of time, shifting only as an individual remains in extended recovery. Consequently, for individuals whose stoppage of substance use is relatively recent, it is possible that their perceptions of others’ stigma remain unchanged.

By examining whether stigma may change with the cessation of substance use associated with SUD, this study provides a more nuanced understanding of the needs of people who use substances as it relates to stigma reduction. Study results suggest that anti-stigma campaigns that target the community perceptions of people with SUD could lead to reductions in stigma of both those who still use and those who may have ceased using substances, but only if campaigns are broadened to encompass the experiences of people who are currently using as well as those who are no longer using substances. Results point to the necessity to design anti-stigma campaigns utilizing the perspectives of individuals currently using substances and individuals that have ceased using substances related to a SUD to capture both of these perspectives. Further, as this study indicates that self-stigma remains higher among those who are still using substances, anti-stigma programs can specifically target the domain of self-stigma (e.g., shame) among those who are still using substances, especially as self-stigma has been associated with difficulties with sustained engagement with treatment and recovery (19, 21).

### 4.1 Limitations and strengths

While this study’s community-based nature is a strength (detailed below), there are accompanying limitations. First, recruitment was undertaken via convenience sampling in the community through leaders and social media, and thus was subject to self-selection bias. Despite these concerns, these recruitment strategies were determined to be the best for ensuring confidentiality and an optimal response rate, as possible, in the community. All data provided are self-reported, including our main independent variable of whether the participant is or is not currently free of substances associated with their SUD; the self-reported nature of the measures is thus subject to possible under-reporting bias. Additionally, there was no way to ascertain how long someone has ceased using substances, which could influence perceptions of stigma from community others. Future research should consider the length of cessation of use. The sample was demographically skewed toward more White, higher income, male, and younger people. Additionally, the two groups, those who currently used substances compared to those who did not, were significantly different among some demographic characteristics. Although demographic characteristics were controlled for in analyses, as appropriate, the generalizability of results beyond this study may be limited. Lastly, the measures of stigma used in this study, while adapted from previously validated measures, were not validated for their adapted versions, which may impact their construct validity. This was further reflected in the measures of stigma having lower internal consistency than expected, although internal consistency remained at adequate levels overall.

Despite these limitations, this study has key strengths. This is one of the first studies in a relatively large community sample to quantitatively examine stigma as it differs between groups of individuals who currently or do not currently use substances associated with their SUD. Understanding how stigma differs with cessation of substance use has enormous practical implications for stigma reduction efforts in helping to support individuals who are no longer using substances to continue in their cessation. Additionally, this study utilized a range of different stigma measures to provide a nuanced examination of stigma domains, given that it is a multidimensional construct involving both self- and other-oriented perceptions.

### 4.2 Future directions

This study provides a foundation for future research and practice to reduce stigma across its various domains and support the recovery process for individuals who are in the process of recovering from SUD. Future research should go beyond cessation to understand how other aspects of recovery beyond stoppage of use are related to stigma. As recovery is not a singular concept (14), stigma domains could vary in their relationship with other facets of recovery, such as reduction in use, length of cessation, or engagement with medication-assisted treatment, and should be studied in more depth to broaden our understanding of how these key elements of recovery are (or are not) related with stigma.

## Data availability statement

The datasets presented in this article are not readily available because of ethical and confidentiality concerns. Information on sensitive topics was collected from individuals who self-identified as using substances (including illegal substances) and from specific counties in Michigan, USA. For any questions or requests regarding the data to be considered, please contact the corresponding author. Requests to access the datasets should be directed to the corresponding author.

## Ethics statement

The studies involving humans were approved by Michigan Department of Health and Human Services, Institutional Review Board for the Protection of Human Research Subjects, and determined as “not human subjects research.” The studies were conducted in accordance with the local legislation and institutional requirements. Written informed consent for participation was not required from the participants or the participants’ legal guardians/next of kin because participants were informed of the purpose, confidentiality, and data use in the online survey, and could choose to participate. Consent was assumed upon participation and submission of the online survey.

## Author contributions

KP: Formal analysis, Writing – original draft, Writing – review & editing. EP: Writing – original draft, Writing – review & editing, Investigation. DN: Project administration, Writing – review & editing, Investigation. ED: Writing – original draft, Writing – review & editing. XW: Writing – original draft, Writing – review & editing. LY: Methodology, Supervision, Writing – review & editing.
